# A homogeneous dataset of polyglutamine and glutamine rich aggregating peptides simulations

**DOI:** 10.1016/j.dib.2021.107109

**Published:** 2021-04-30

**Authors:** Exequiel E. Barrera, Sergio Pantano, Francesco Zonta

**Affiliations:** a Instituto de Histología y Embriología (IHEM) - Consejo Nacional de Investigaciones Científicas y Técnicas (CONICET), CC56, Universidad Nacional de Cuyo (UNCuyo), Mendoza, Argentina; b Biomolecular Simulations Group, Institut Pasteur de Montevideo, Mataojo 2020, CP 11400 Montevideo, Uruguay; c Shanghai Institute for Advanced Immunochemical Studies, ShanghaiTech University, Shanghai 201210, China

**Keywords:** SIRAH, Coarse-grained simulation, Soluble oligomer, polyQ, Q-rich, Aggregation, Oligomerization, Molecular dynamics

## Abstract

This dataset contains a collection of molecular dynamics (MD) simulations of polyglutamine (polyQ) and glutamine-rich (Q-rich) peptides in the multi-microsecond timescale. Primary data from coarse-grained simulations performed using the SIRAH force field has been processed to provide fully atomistic coordinates. The dataset encloses MD trajectories of polyQs of 4 (Q4), 11 (Q11), and 36 (Q36) amino acids long. In the case of Q11, simulations in presence of Q5 and QEQQQ peptides, which modulate aggregation, are also included. The dataset also comprises MD trajectories of the gliadin related p31-43 peptide, and Insulin's C-peptide at pH=7 and pH=3.2, which constitute examples of Q-rich and Q-poor aggregating peptides. The dataset grants molecular insights on the role of glutamines in spontaneous and unbiased ab-initio aggregation of a series of peptides using a homogeneous set of simulations [1]. The trajectory files are provided in Protein Data Bank (PDB) format containing the Cartesian coordinates of all heavy atoms in the aggregating peptides. Further analyses of the trajectories can be performed directly using any molecular visualization/analysis software suites.

## Specifications Table

**Table:** 

Subject	Biological Sciences.
Specific subject area	Protein Biophysics. Molecular dynamics simulations of aggregating peptides.
Type of data	Secondary Data. Molecular dynamics trajectories of multiple peptide systems.
How data were acquired	Hardware: CPU (Intel Core i7-5930K, 3.5 GHz) accelerated with a TitanX GPU. Software: Gromacs 2018.4 using the SIRAH 2.0 force-field for performing MD simulations and SIRAH Tools, along with AmberTools 2018 and Amber14SB force-field implemented in VMD 1.9.3 for backmapping.
Data format	Filtered.
Parameters for data collection	MD simulations were performed at 300K and 1 Bar for multiple microseconds. Full details of all simulations are reported in Table 1.
Description of data collection	Raw molecular dynamics data at coarse-grained level was filtered to maintain one every ten steps and and protein's heavy atoms were backmapped using SIRAH Tools. Simulation frames are reported every 1 ns of simulation.
Data source location	Primary Data was collected at the Uruguayan Center for Supercomputation (ClusterUY).
Data accessibility	Repository name: Mendeley Data Direct URL to data: https://data.mendeley.com/datasets/2tmsbchh42/2 Instructions for accessing these data: Data is freely accessible.
Related research article	The primary data source consists of a set of coarse-grained MD simulations. They are described in the associated manuscript “Dissecting the role of glutamine in seeding peptide aggregation” by E. E. Barrera, F. Zonta, and S. Pantano, Computational and Structural Biotechnology Journal, 2021, DOI: https://doi.org/10.1016/j.csbj.2021.02.014

## Value of the Data

• Homogeneous sets of simulations on different aggregating peptides on multimicroseconds timescale are very rare in the literature. Analysis of this dataset can provide valuable insights obviating the lengthy process of generating the data from the scratch.
• Data of interest to computational biophysicist/biochemists studying peptide aggregation.
• Molecular coordinates can be read/analyzed with standard software for structural biology or molecular visualization.

## 1 Data Description

The dataset is deposited on Mendeley data with the doi: 10.17632/2tmsbchh42.2. It contains two .zip files (one for the polyglutamine peptides and another for the Q-rich peptides) enclosing separated files for each peptide trajectory. The peptide composition and specifics of each system, and name of individual data trajectories are reported in Table 1. This dataset contains eight files of molecular trajectories of different peptides in Protein Data Bank (pdb) format that can be visualized/analyzed with standard molecular visualization/simulation programs.

**Table 1:** Summary of the systems simulated.

Peptide	Monomers in the box	Box size (nm)	Peptide concentration (mM)	Protonation state in the termini	Length of the Simulations (µs)	Name of the file in Mendeley data
Q4	27	11.5 (cubic)	29.4	neutral	3	Q4_agg_5us.pdb
Q11	10	13.5 (cubic)	6.7	neutral	5	Q11_agg_5us.pdb
Q11 + Q5	20	13.5 (cubic)	13.4	neutral	5	Q11-QQQQQ_agg_5us.pdb
Q11 + QEQQQ	20	13.5 (cubic)	13.4	neutral	5	Q11-QEQQQ_agg_5us.pdb
Q36	3	13.5 (cubic)	2.1	neutral	5	Q36_agg_5us.pdb
p31-43	50	23 × 22 × 19 (octahedral)	8.4	neutral	5	p31-43_agg_5us.pdb
C-peptide	30	23 × 22 × 19 (octahedral)	5.1	zwitterionic	5	C-peptide_agg_pH7_5us.pdb
C-peptide	30	23 × 22 × 19 (octahedral)	5.1	N-terminal (+) C-terminal (neutral)	5	C-peptide_agg_pH3.2_5us.pdb

## 2 Experimental Design, Materials and Methods

### 2.1 Primary data

A detailed description of the protocol followed to generate the primary data is reported in the associated paper [1]. Briefly, for each system we started from fully atomistic peptide copies that were uniformly distributed in simulation boxes listed in Table 1. Systems were mapped to coarse-grain using SIRAH Tools [2], and solvated. In the simulations of the C-peptide at pH = 7 and pH = 3.2, KCl ions were added to a concentration of 150 mM. MD simulations were performed in the NPT ensemble at 300 K and 1 atm using the SIRAH force field version 2.0 [3] using GROMACS 2018.4 as simulation engine [4].

### 2.2 Secondary data

The secondary data consists of the trajectories of the peptides reported in Table 1 backmapped to fully atomistic representation. This will allow to interested scientist to run straightforwardly further analyses using standard simulation/structural biology tools obviating the significant computational cost associated to the generation of the data and facilitate the interpretation of the coarse-grained representation to non-experts. Backmapping was performed using SIRAH Tools [2]. To this aim we used a tcl script included in the distribution that can be loaded on the popular molecular visualization software named VMD 1.9.3 [5] Once the coarse-grained trajectories are loaded, they are processed one frame at the time. Since the simplified SIRAH representation preserves the position of a few atoms in each residue, individual simulation frames were taken separately and missing atoms were first added using internal coordinates residue by residue. The reconstructed molecules were then loaded to the tleap module of Amber18 [6] to generate individual topology and coordinates. Subsequently, these coordinates underwent an all-atoms energy minimization in vacuum with a cut off of 1.2 nm using the sander module of Amber18 and the Amber14SB force field [7]. We performed 50 steps of energy minimization using the steepest descent algorithm followed by 100 steps using conjugated gradient Finally, atomistic structures were concatenated and saved into one single trajectory files. Each of the trajectory files listed in Table 1 contains one frame per ns. To preserve the portability of the dataset, only the trajectories containing the heavy atoms of the peptides are reported in the database. It is important to notice that the above-described process is integrated in SIRAH tools and executed with a command line from the VMD console.

## Ethics Statement

Not applicable.

## CRediT Author Statement

**E.E. Barrera:** Conception and design of study, Acquisition of data, Analysis and/or interpretation of data, Drafting the manuscript, Revising the manuscript critically for important intellectual content; **S. Pantano:** Conception and design of study, Acquisition of data, Analysis and/or interpretation of data, Drafting the manuscript, Revising the manuscript critically for important intellectual content; **F. Zonta:** Analysis and/or interpretation of data, Drafting the manuscript, Revising the manuscript critically for important intellectual content.

## Declaration of Competing Interest

The authors declare that they have no known competing financial interests or personal relationships, which have or could be perceived to have influenced the work reported in this article.
